# Treatment of a life-threatening dapsone intoxication

**DOI:** 10.1016/j.toxrep.2023.10.006

**Published:** 2023-10-13

**Authors:** M.H. Veerman, T. van Gelder, R. Sneijder, C. Bethlehem

**Affiliations:** a Department of Hospital Pharmacy, Erasmus University Medical Center, Rotterdam, the Netherlands; b Department of Intensive Care, ADRZ Medical Center, Goes, the Netherlands

**Keywords:** Pharmacokinetics, Toxicology, Poisoning, Dapsone, Methotrexate, Methemoglobinemia, Hepatitis

## Abstract

The case report describes a case of a severe dapsone (more than 200 tablets dapsone 100 mg) and mild methotrexate intoxication (10 tablets methotrexate 10 mg) as an attempt to commit suicide, resulting in severe cyanosis with elevation in methemoglobin concentration, treated with methylene blue, ascorbic acid, folinic acid, multidose activated charcoal and hemodialysis. Measurements of blood gases, dapsone and methotrexate levels were performed. Furthermore a hepatitis, pulmonary artery thrombus and a strange taste sensation were diagnosed, probably related to dapsone. The patient recovered and was discharged from hospital after five days. Acute intoxication from excessive dapsone intake is uncommon and clear treatment guidelines are lacking. We here report the treatment modalities as a result of a dapsone intoxication, including the effects on the overall condition of the patient.

## 1 Introduction

As an intentional suicide attempt, a patient ingested a relative’s medication during nighttime. Soon after intake of the medication he started vomiting. In the morning, the patient was found by the relative, in a confused state and with a poor skin color. The general practitioner who visited the patient at home diagnosed central cyanosis and called an ambulance. When the ambulance arrived, the oxygen saturation was 54 % and the patient received supplementary oxygen via a non-rebreathing mask (NRM) at a flow rate of 15 liters/minute. He was hemodynamically stable and had no dyspnea. Other than nausea and a vague feeling in the upper abdomen, he had no other complaints.

## 2 Case report

The anamnesis, physical and laboratory examination after admission are presented in Table 1. The patient was transparent about his suicide attempt by taking medicines, but wasn’t able to tell the hospital team which medicines or how many he had taken. The relative’s medication to which he had access was: methotrexate 10 mg, dapsone 100 mg, folic acid 5 mg, omeprazole 20 mg, ferrous fumarate 200 mg, diclofenac 50 mg, metoprolol 50 mg and hydroquinine 100 mg. The cyanosis, in combination with methemoglobinemia, led to the suspicion of a dapsone intoxication (Table 2).

**Table 1:** The anamnesis, physical and laboratory examination after admission.

Patient	middle-aged Caucasian man
BMI	normal
Smoker	yes, 1 pack per week
Prescribed medications	no
Glucose-6-phosphate dehydrogenase deficiency (G6PD)	no
Allergies	no
Medical history	gastric ulcer, varicose veins
Psychiatric history	no
	
Airway	free
Thorax x-ray	normal
Saturation with NRM 15 L/min	95 %#
Cyanosis	central and peripheral
Blood pressure	normal
ECG	normal
Glasgow coma scale	maximum
Pain	no
	
Hemoglobin	normal
Hematocrit	normal
Renal function	normal
Methemoglobinemia	**present (39.9 %)**
Glucose	elevated (9.3 mmol/L)
Lactate	elevated (5.9 mmol/L)
Leucocytes	elevated (27.0 × 10^9/L)
Paracetamol	not detectable

# Be aware: in cases of increased MetHb fraction, pulse oximeter values trend toward 85 %, underestimating the actual oxygen saturation [1], [2].

**Table 2:** Chemical, pharmacokinetic and clinical characteristics of dapsone [3], [4].

Dapsone	Structure	
	Adult daily dose (mg)	50–400
	Molecular weight (Da)	248
Absorption	Oral availability (%)	∼ 100 %
	Cmax (h)	4–8
Distribution	Plasmabinding (%)	70–90
	Volume of distribution (L/kg)	1.5
	Enterohepatic circulation	yes
Elimination	Routes of metabolism	hydroxylation, acetylation
	Main metabolites	monoacetyldapsone (MADDS), dapsone hydroxylamine (DDS-NHOH), mono acetyl dapsone hydroxylamine (MADDS-NHOH)*
	Half life (h)	10–50 (average 28)
	Urinary excretion (%)	70–80 (10–20 % unchanged)
Lab monitoring	Therapeutic serum concentration (mg/L)	0.5–5.0
	Toxic concentration (mg/L)	> 10
Clinical monitoring	Manifestations of acute intoxication	methemoglobinemia, hemolysis, vomiting, cyanosis, tachypneu, tachycardia, altered or depressed mental status or seizures
	Contraindications	hypersensitivity to dapsone, glucose-6-phosphate dehydrogenase (G6PD) deficiency, methylene blue dose > 4 mg/kg in total; ascorbic acid dose> 4 g/day

* DDS-NHOH and MADDS-NHOH are responsible for hemotoxic reactions.

Due to the methemoglobinemia, methylene blue and ascorbic acid was started and oxygen suppletion was continued. Multiple-Dose Activated Charcoal (MDAC) and laxatives were prescribed to prevent the absorption from the gastrointestinal tract and enhance the elimination of dapsone as it interferes with the enterohepatic circulation. Folinic acid was prescribed as an antidote to methotrexate and pantoprazole was prescribed for gastric protection.

During hospitalization the patient was hydrated and the renal function and diuresis remained stable (≥ 82 ml/min/1.73 m^2^). The glucose and lactate levels normalized within 24 h.

**Fig. 1:**
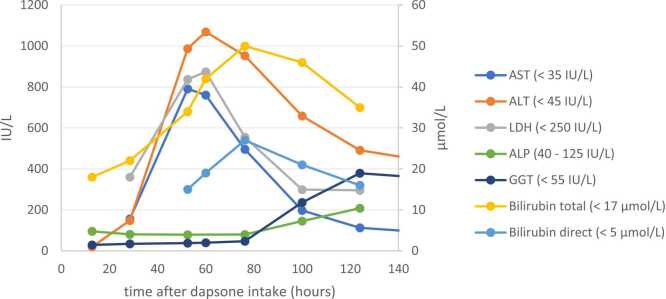
Changes in liver enzymes over time.

The liver enzymes were rising and returned to normal values, see Fig. 1 [5]. Clinically the patient was doing well, but he was hypoxic (saturation 89 % in room air) without symptoms. A pulmonary artery thrombus was diagnosed with computed tomography (CT) and anticoagulant medication was prescribed [6], [7]. The patient risk factors for pulmonary embolism were: smoking, admission to the intensive care, immobilization and a central venous catheter line. Furthermore, while eating, the patient mentioned a strange taste sensation [8].

Blood samples for measurement of methotrexate and dapsone concentrations were collected at different times. There was a delay time between taking the dapsone blood samples and getting the results. Treatment was therefore based on the clinical symptoms and the assumed amount of medicines taken. The methotrexate intoxication was mild, without clinical symptoms and was therefore not discussed in detail.

On day 1 after admission, the relative reported that the following medicines may have been ingested: 1 box of metoprolol 50 mg, 2 boxes of ferrous fumarate 200 mg, 1 vial of dapsone 100 mg (> 200 tablets) and 10 tablets of methotrexate 10 mg. Clinically, there was no suspicion of severe metoprolol or ferrous fumarate intoxication. The patient was hemodynamically stable, the ECG was normal, there was no metabolic acidosis and the gastrointestinal complaints resolved quickly after admission. The high dose of dapsone taken by this patient could be lethal. There is limited data on the clinical effect of extracorporeal treatments on dapsone elimination. In view of the high volume of distribution, high degree of protein binding and minimal excretion of unchanged drug the usefulness of extracorporeal treatments to accelerate the clearance of dapsone is questionable. In the literature however there is some evidence that hemodialysis could accelerate the clearance of dapsone [9], [10]. Due to the excessive dose of dapsone taken, hemodialysis was performed in this case. The patient underwent hemodialysis without problems. The monitoring and treatment regimens are presented in Table 3.

**Table 3:** Dapsone and MTX monitoring and treatment regimes.

Time after intake (h)	DPS level (mg/L)	Activated charcoal (g)	Hemodialysis	MetHb	Methylene blue and ascorbic acid	Oxygen 15 liter/min via NRM	MTX level	Folinic acid
12.8	31.5			39.9		ongoing	0.33	
14					2 mg/kg + 1000 mg			4dd15 mg
16		50		8.7				
28.5	24			21.5			0.12	
32.5					2 mg/kg + 1000 mg			
34.5	20.9		start					
36		25						
38.3	16.8		stop					
39.6				8.1				
40		25						
44		25						
48		25						stop
52		25						
55.8	6.9		start					
56		25						
56.5					2 mg/kg + 1000 mg			
60		25	stop	2.2				
64		25						
68		25						
76.3				3.3				
85.3				3.1		stop		

## 3 Discussion

Dapsone is contraindicated in case of severe G6PD deficiency and/or severe anemia. Caution is advised in case of liver function disorders. Routine blood monitoring is the recommended standard care during the course of dapsone therapy. In this case there was no anemia or suspicion of G6PD deficiency. The elevated liver enzymes in this case were likely caused by the intoxication and not present before presentation.

**Fig. 2:**
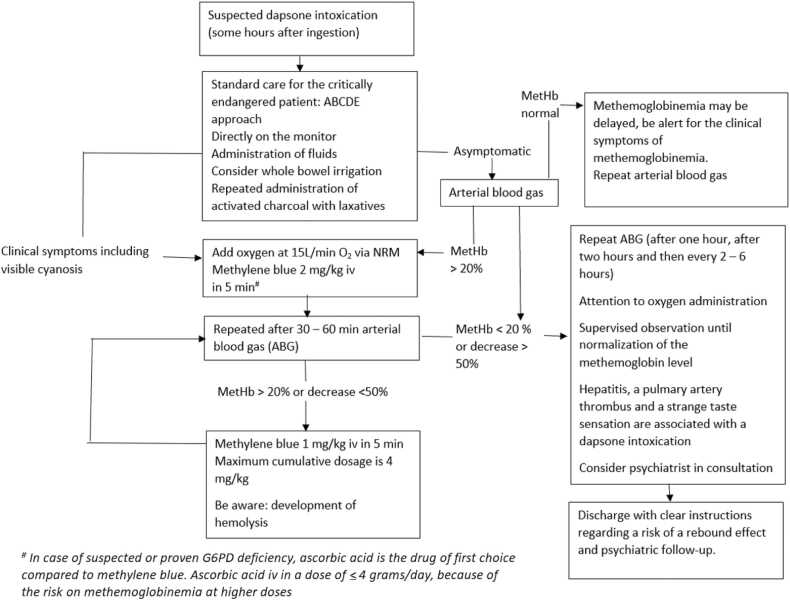
Flowchart treatment dapsone intoxication.

The added value of hemodialysis on the clearance of dapsone to the administration of MDAC was limited in this patient, who had a good renal function and no hypoalbuminemia. Therefore hemodialysis is also not recommended for the treatment of a dapsone intoxication (Fig. 2). The literature on the preferred treatment of dapsone intoxication is very limited. Treatment guidelines are largely based on a few reported cases and some theoretical pharmacological considerations. The hemodialysis applied in this case was more based on the idea that it might be of benefit, than based on evidence based guidelines.

The use of activated charcoal, methylene blue and ascorbic acid was effective. In this case methylene blue with ascorbic acid is prescribed to treat methemoglobinemia. But methylene blue monotherapy is the treatment of choice for patients, other than those with suspected or proven G6PD deficiency, in which cases ascorbic acid monotherapy is used instead. Methylene blue acts within 30–60 min, treatment with ascorbic acid appears to take one to three days depending on the methemoglobinemia level. Dapsone can produce a rebound methemoglobinemia, as in our case, in which methemoglobin levels increase 4–12 h after successful methylene blue therapy.

The measured dapsone level was lower than the predicted dapsone level based on the assumed number of tablets taken. Possibly this is due to the vomiting immediately after intake, less intake than assumed, a slower absorption and/or a different metabolism pattern (only the dapsone level has been measured, the analytic method was not validated for dapsone metabolites). Considering the course of the dapsone levels, vomiting after intake and/or less intake are the most plausible.

The patient recovered and was discharged from hospital after five days.

## 4 Conclusion

A dapsone intoxication can be life-threatening due to the development of severe methemoglobinemia. A potential severe dapsone intoxication requires close clinical and laboratory monitoring. May this case, and the flowchart shown in Fig. 2, help in the treatment of future dapsone intoxications.

## Declaration of Competing Interest

The authors declare that they have no known competing financial interests or personal relationships that could have appeared to influence the work reported in this paper.

## Data Availability

Data will be made available on request.
